# Fused Filament Fabrication of a Dynamically Crosslinked Network Derived from Commodity Thermoplastics

**DOI:** 10.1021/acsapm.2c00340

**Published:** 2022-05-10

**Authors:** Goutam Prasanna Kar, Xueyan Lin, Eugene Michael Terentjev

**Affiliations:** Cavendish Laboratory, University of Cambridge, JJ Thomson Avenue, Cambridge CB3 0HE, U.K.

**Keywords:** additive manufacturing, shape-memory polymers, epoxy−anhydride crosslinking, dynamic covalent chemistry, vitrimer, thermoplastic recycling

## Abstract

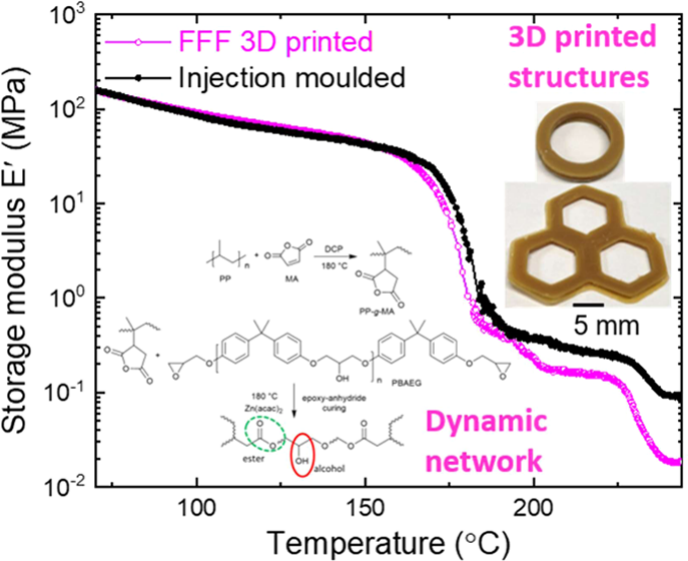

A massive carbon footprint is associated with the ubiquitous use of plastics and their afterlife. Greenhouse gas (GHG) emissions from plastics are rising and increasingly consuming the global “carbon budget”. It is, hence, paramount to implement an effective strategy to reclaim postconsumer plastic as feedstock for technologically innovative materials. Credible opportunity is offered by advances in materials chemistry and catalysis. Here, we demonstrate that by dynamically crosslinking thermoplastic polyolefins, commodity plastics can be upcycled into technically superior and economically competitive materials. A broadly applicable crosslinking strategy has been applied to polymers containing solely carbon–carbon and carbon–hydrogen bonds, initially by maleic anhydride functionalization, followed by epoxy–anhydride curing. These dynamic networks show a distinct rubber modulus above the melting transition. We demonstrate that sustainability and performance do not have to be mutually exclusive. The dynamic network can be extruded into a continuous filament to be in three-dimensional (3D) printing of complex objects, which retain the mechanical integrity of vitrimers. Being covalently crosslinked, these networks show a thermally triggered shape-memory response, with 90% recovery of a programmed shape. This study opens up the possibility of reclaiming recycled thermoplastics by imparting performance, sustainability, and technological advances to the reprocessed plastic.

## Introduction

Fossil fuel-based feedstock is the building block of the majority of plastics today.^1^ Since the last four decades, plastic production has quadrupled. Ever-increasing anthropogenic greenhouse gas (GHG) emissions are associated with proliferating plastic production, endangering the environment. Global life-cycle GHG emissions of plastic were 1.7 Gt CO_2_-equivalent (CO_2_e) in 2015 and are projected to reach 6.5 Gt CO_2_e by 2050. It is estimated that if the current trend in GHG emissions from plastics is continued, it will consume 15% of the global “carbon budget” by 2050, thus presenting a critical concern to the global effort to curb total anthropogenic carbon emission.^2^

Production of carbon-intensive virgin polymers can be significantly reduced by recycling after the service life of commodity plastics.^3^ Globally, over 150 million tons of plastic are disposed of annually, and only about 10% are being recycled. Megatons of this plastic can be an untapped resource and a valuable feedstock.^4^ Conventional mechanical recycling is a downward spiral in terms of technical and monetary values caused by deteriorating mechanical properties due to the incomplete purification. Energy recovery as an end-of-life treatment such as incineration leads to the release of toxins and carbon emissions. It is thus paramount to develop an efficient strategy to enable polymers to remain inherently recyclable without compromising their material properties. In principle, it is possible to chemically tailor the functionality of the polymer backbone to remake high-valued materials with minimal loss of performance, thus enabling re-entry into the functional life cycle; such a feat is the hallmark of the cradle-to-cradle life cycle of plastics having a profound impact in mitigating GHG emissions.^5,6^

Canonically, synthetic polymers are classified into two distinct families: thermoplastics and thermosets. Held together by physical intermolecular forces, thermoplastics are simply entangled polymer chains; although lightweight, tough, and reprocessable, they are often soluble and cannot preserve mechanical integrity at elevated temperatures. Thermosets, on the other hand, are covalently crosslinked networks and thus tough, insoluble, and withstand high temperatures. However, being permanently crosslinked, these materials are “petrified” after synthesis and, hence, cannot be reprocessed and repurposed. The fixed geometry of injection molding restricts these high-performing thermoset materials to a limited set of shapes; they cannot be recycled and face detrimental end-of-life treatments such as incineration.^3^

The recent renaissance in thermoset chemistry has challenged this dogma by underpinning a molecular mechanism of bond exchange, leading to a characteristic elastic–plastic transition enabled by dynamic crosslinking. Dynamic covalent chemistry has been applied to fundamentally alter the viscoelastic behavior of thermosets.^7−9^ When the covalent bond exchange is triggered by external stimuli, the crosslinks rearrange among themselves, facilitating plastic flow under stress (which is a very different process from the viscous flow of a melt). In the absence of a trigger, the dynamic network behaves as a traditional thermoset. Several bond exchange reactions have been explored to impart dynamic nature to the polymer network, such as transesterification,^10^ transamidation of vinylogous urethanes,^11^ olefin metathesis,^12^ disulfide metathesis,^13^ dioxaborolane metathesis,^14,15^ thiol-disulfide exchange,^16^ and transalkylation.^17^ While the viscosity of thermoplastics is governed by monomer friction, the rate of bond exchange in the dynamically crosslinked network dictates the rate of its plastic flow at elevated temperatures. The Arrhenius thermally activated dependence of this rate enables reprocessing these materials like vitreous silica; hence, these types of materials were named *vitrimers* by Leibler and co-workers.^18^ This bridging of the thermomechanical behavior between thermoplastics and thermosets results in a range of smart materials. Advanced polymer characteristics, such as strong welding,^19^ shape-memory response,^20−22^ and self-healing,^13,16,23^ have been demonstrated with first-generation thermoplastic vitrimers of different kinds. Given the intensive ongoing research, it is expected for dynamic covalent chemistry to produce a large library of smart materials enabling a multitude of current applications in aerospace, automobile, biomedical, flexible electronics, and other industrial fields.

Here, we demonstrate a broadly applicable strategy of dynamic crosslinking in polymers containing solely carbon–carbon and carbon–hydrogen bonds. Thermoplastic polyolefin (TPO) such as polypropylene (PP) and polyethylene (PE) comprising the C–C backbone constitutes 75% of polymers produced globally.^1,14^ The absence of functional groups in TPO makes them chemically inert, also making functionalization difficult. To address this challenge, Leibler et al.^14^ have formed TPO-based vitrimers crosslinked by dioxaborolane; this approach was repeated in more recent work by Yang et al.^24^ and Maaz et al.^25^ However, the borolane bond exchange has very low activation energy, and the resulting materials would creep at high temperatures. Exploring other crosslinking options, He et al. used the imine bond exchange with an activation energy of ca. 57 kJ/mol,^26^ while Saed et al. used the thiol–thioester bond exchange with an activation energy of ca. 110 kJ/mol.^27^ In an attempt to form a vitrimer with an even higher thermal stability, we have used the diepoxy crosslinking of functionalized TPO, looking for a higher activation energy of transesterification bond exchange, reported as ca.124 kJ/mol.^10^

Here, we continued to work with PP as a model system and functionalized it by grafting the maleic anhydride (MA) to enable robust epoxy-ester bonding. To help with transesterification, we selected a diepoxy oligomer with an added hydroxyl group to demonstrate the production of a dynamic network and remarkable changes in its mechanical response to a thermal stimulus.

The transformation of commodity thermoplastics into smart polymer materials can only be viable if the method is compatible with the current settings of the polymer processing industry. In this context, a solvent-free melt compounder has been employed. Over the years, melt compounders have emerged in various forms that have been coupled to other postprocessing steps, such as melt blowing and injection molding. Additive manufacturing has added another dimension to material design,^24^ when functional polymeric materials are extruded as a continuous filament from a melt compounder, and then served to a three-dimensional (3D) printer in the fused filament fabrication (FFF) mode to produce digitally encoded structure on demand.^28,29^ Here, to demonstrate this process, the vitrimer derived from commodity plastics was manufactured into complex objects with high-dimensional precession. After that, we verified whether the material properties were preserved after the FFF 3D-printing process. This workflow will have an impact on producing technically innovative and economically competitive materials from a generic commodity plastic feedstock, a key milestone toward the reduction of GHG emissions of plastics and the increase of their multiple uses. Thermoplastics can also be 3D-printed, but vitrimers have a strong welding capacity to allow the subsequent assembly of 3D-printed parts, a property not available to thermoplastics. Being covalently crosslinked, semicrystalline plastic vitrimers will also possess a very strong shape-memory response, which we particularly explore in this work.

## Experimental Section

### Materials

Polypropylene (PP) with a melt flow index of 34 g/10 min was procured from INEOS Olefins & Polymers, USA. Maleic anhydride (MA), dicumyl peroxide (DCP), poly(bisphenol A-*co*-epichlorohydrin) glycidyl end-capped (PBAEG) with a *M*_n_ of ≈1075, zinc acetylacetonate hydrate (Zn(acac)_2_), and xylene solvent were supplied by Sigma Aldrich (Merck).

### Reactive Extrusion

**Figure 1:**
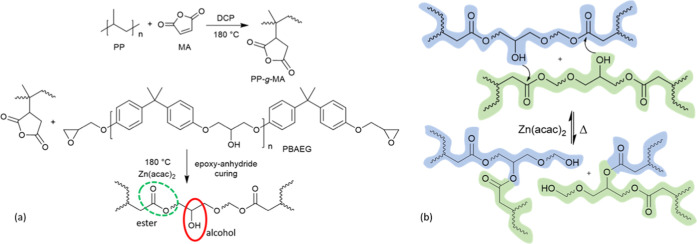
Synthesis of a dynamically crosslinked PP network. (a) Two-step functionalization of PP to introduce dynamic crosslinking: in the first step, anhydride functional groups are grafted onto the PP backbone through a free-radical reaction. Zn(acac)_2_-catalyzed epoxy–anhydride curing is performed in the second step. (b) Thermally activated bond exchange between ester and alcohol functional groups (transesterification) in the dynamic network.

A dynamically crosslinked polyolefin network was synthesized through reactive extrusion on a laboratory-scale twin-screw extruder (HAAKE MiniLab 3, ThermoFisher) equipped with a recirculation channel. The whole process was carried out at 180 °C, with a screw speed of 100 rpm under N_2_ flow. First, the required amount (4–5 g) of stock thermoplastic, MA (5 wt %), and the DCP (0.1 wt %) initiator were fed with the aid of a pneumatic press. After 10 min of circulation, we verified the saturation of MA grafting, and the difunctional epoxy crosslinking PBAEG with the Zn(acac)_2_ (2.0 wt %) catalyst^30^ was added into the extruder. After another 10 min, i.e., with a total residence time of 20–22 min, the crosslinked network was extruded. The resulting vitrimer was postprocessed with a laboratory injection molding (MiniJet, ThermoFisher) at 210 °C to obtain a specimen with the required dimensions. The uniform FFF filament with a diameter of ca. 1.75 mm was prepared by directly extruding the vitrimer through a custom-made nozzle with a diameter of 2 mm. The scheme of the reactions is given in Figure 1.

### Gel Fraction

Samples of crosslinked vitrimers (weight = *W*_*i*_) were immersed in xylene at 120 °C for 24 h, with a frequently changing solvent. The residual solid was dried at 120 °C in a vacuum oven (typically for 6 h) until a constant weight (*W_f_*) was achieved. Gel fraction (%) is measured as (*W*_*f*_/*W*_*i*_) × 100.

### Melt Flow Index

An essential rheological characteristic of plastics is their melt flow index (MFI), which determines the regime and parameters of the processing flow. The ASTM D1238 protocol describes the MFI measurement, which we have followed in this work. The PP and its derivatives fall into a category that is tested at 230 °C under a driving pressure of Δ*P* = 0.3 MPa (achieved by supplying a 2.16 kg load across the 9.5 mm cylinder bore). The plastic flow is measured through a standard die of *d* = 2 mm diameter and *L* = 8 mm length, and the resulting MFI is measured in units of grams per 10 min. Assuming Poiseuille flow, the volumetric flow rate Q is given by the expression
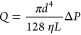
where η is the flow viscosity. Therefore, measuring the MFI in units of mass per unit time is equivalent to measuring the viscosity η of the flowing polymer.

### Fourier Transform Infrared (FT-IR)

**Figure 2:**
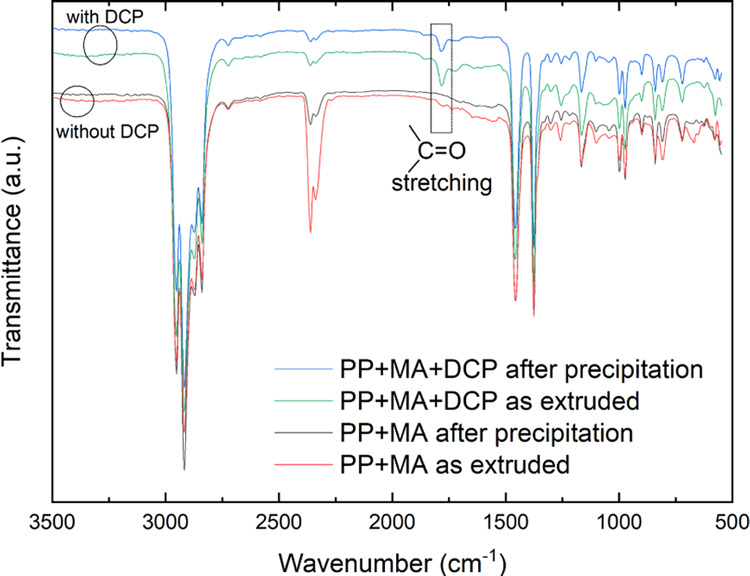
FT-IR spectra of functionalized PP. The C=O absorbance band is highlighted at 1785 cm^–1^. Extrusion performed without a free radical initiator DCP does not yield covalent attachment between MA and PP. Hence, free MA readily dissolves in acetone during precipitation, and the corresponding C=O stretching band disappears. While reactive extrusion is done with DCP, MA is covalently bonded onto PP, and the C=O stretching band is retained after precipitation.

MA grafting onto PP was confirmed by FT-IR. A compression-molded specimen was characterized with a PerkinElmer Spectrum 100 FT-IR spectrometer in ATR mode, as shown in Figure 2.

### Titration

The percentage of MA grafting was quantified by titration. Typically, 0.5 g of MA-g-PP was dissolved in 50 mL of xylene at 120 °C. A few drops of water were added to the mixture. Water hydrolyzes maleic anhydride into a carboxylic acid group, which was titrated with alcoholic KOH (0.1 N) using 1 wt % bromothymol blue (in dimethylformamide) as an indicator. As the indicator was added to the mixture (acidic solution), it appeared yellowish. The titration endpoint was identified by the appearance of a faint blue color. The amount of KOH (0.1 N) was noted, and the titration was performed in triplicate. The percent grafting was estimated by the relation as
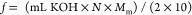
where the normality, *N* = 0.1, and *M*_m_ = 100 is the molecular weight of the grafted MA residue.

### Differential Scanning Calorimetry (DSC)

**Figure 3:**
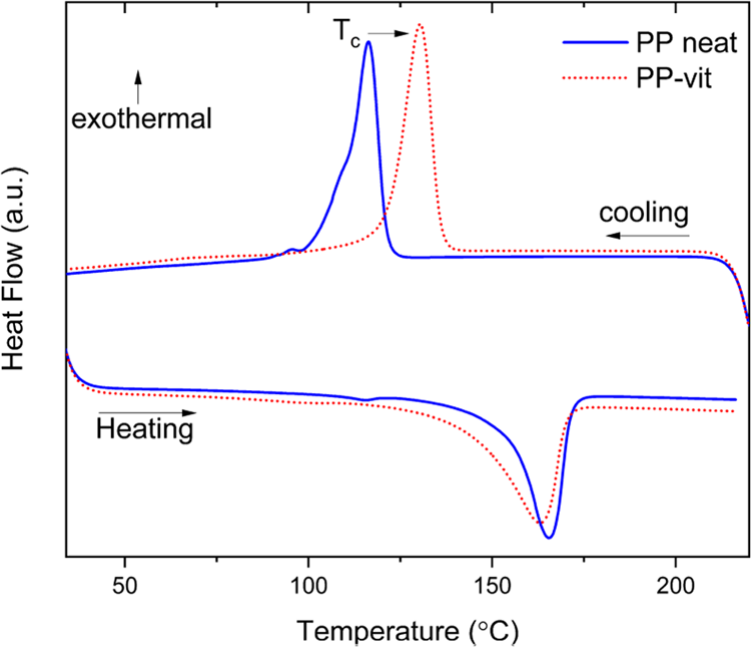
DSC thermogram of vitrimers and its thermoplastic PP precursor. Melting (*T*_m_) and crystallization (*T*_c_) transitions are separated by the usual hysteresis.

Melting (*T*_m_) and crystallization (*T*_c_) transition of the polymers were observed through a DSC4000 from PerkinElmer. Typically, 5–10 mg of dried polymers was heated to 220 °C and maintained isothermally for 5 min to erase the thermal history of the specimen. It was then cooled down to 50 °C. *T*_c_ was observed in this cooling step. A subsequent reheating to 220 °C revealed the *T*_m_ (see Figure 3). All of the heating and cooling cycles were performed at 10 °C/min under N_2_ flow. The percent crystallinity (χ_c_) was calculated as^31^
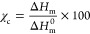
where Δ*H*_m_ is the heat of fusion of the polymer calculated from the area under the melting endotherm (2nd heating) and Δ*H*_m_^0^ is the standard heat of fusion of theoretically calculated 100% crystalline polymer. Δ*H*_m_^0^ for PP was 209 J/g.^32^

### Tensile Testing (Stress–Strain)

**Figure 4:**
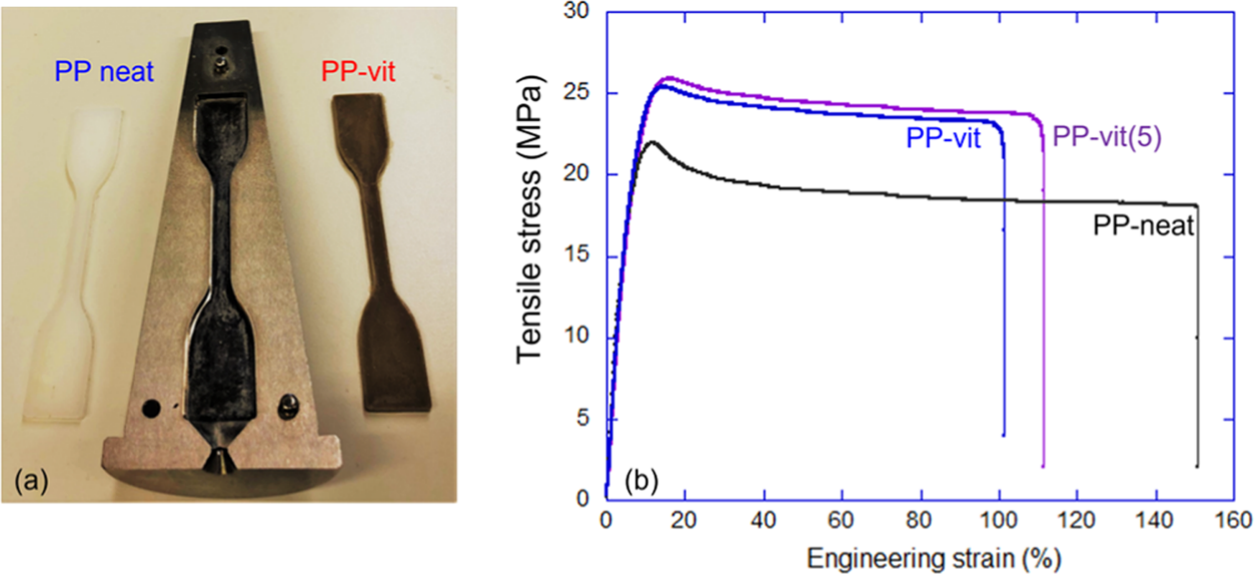
Mechanical properties of the PP vitrimer. (a) ASTM D638 injection mold of the neat PP thermoplastic and the PP-vit, and (b) stress–strain behavior of the dynamic vitrimer network (two variants: freshly crosslinked and after five cycles of recycling), and its thermoplastic PP precursor. Tensile strength and extension were measured at room temperature with a crosshead speed of 5 mm/min as in the test specification.

Room temperature stress–strain behavior was observed on a Tinius Olsen 1ST universal testing machine mounted with a 2kN cell, with a fixed crosshead speed of 5 mm/min. ASTM D638 injection mold was used to prepare the tensile specimen (see Figure 4a).

### Dynamic Mechanical Analysis (DMA)

**Figure 5:**
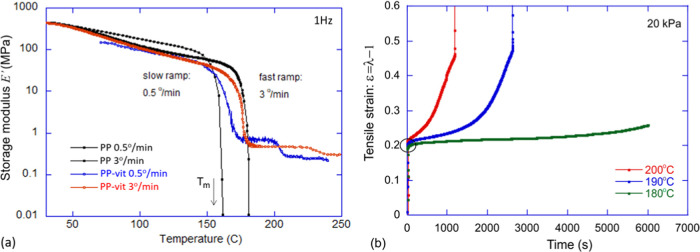
Dynamic mechanical analysis of PP-vit and PP. (a) Evolution of the linear dynamic modulus *E*′ of PP-vit and their original PP thermoplastic with temperature. A slower heating rate (0.5 °C min^–1^) shows the equilibrium melting temperature, which matches the one obtained by DSC. (b) “Iso-stress” creep of the dynamically crosslinked network. An instantaneous tensile stress of 20 kPa is applied to a rectangular specimen at the equilibrated temperature in the rubber regime. At higher temperatures, the bond exchange rate is faster, leading to an enhanced creep rate and hence the early break. At 180 °C, the material responds rubber-elastically, showing very little creep for at least an hour.

Thermomechanical properties were measured with a DMA 850 (TA Instruments). Prior to the test, the samples were kept under vacuum for 5 h at 100 °C. For the measurement of temperature-dependent dynamic modulus, a rectangular specimen of dimension ≈15 mm × 5 mm × 0.9 was placed between the clamps of DMA in tensile mode and equilibrated at 50 °C before starting the experiment. The evolution of the dynamic modulus was observed at a constant frequency of 1 Hz with 0.01% strain in the temperature range of 50–240 °C with a heating rate of 3 °C min^–1^. A much slower heating rate of 0.5 °C min^–1^ was also examined to observe the equilibrium melting transition (see Figure 5a).

### “Iso-stress” Creep Testing

The plastic flow (creep) behavior of the dynamically crosslinked network was observed beyond the melting transition. The rectangular specimen was placed in the DMA clamps in tensile mode and equilibrated at the probe temperature for 5 min. An instantaneous stress of 20 kPa was applied and was kept constant throughout the experiment. The plastic creep of the PP vitrimer was tested at three different temperatures (180, 190, and 200 °C). The evolution of strain with time is represented for each network (see Figure 5b).

### Fused Filament Fabrication (FFF)

Filaments with a diameter of ca. 1.75 mm were extruded from the twin-screw compounder after the crosslinking step was completed. To maintain a uniform diameter, the extrusion speed was reduced to 40 rpm, while the reactive compounding was carried out at 100 rpm. The slow and steady extrusion of the filament ensures a uniform diameter for the whole length of the filament. Fused filament fabrication of the dynamically crosslinked networks was performed in a commercial 3D printer (Hyrel 3D) with a print head MK-250, at a printing head speed of 10 mm/s, a layered height of 0.1 mm, and 100% infill. FFF extrusion printing was performed with a nozzle with a diameter of 0.55 mm at 240 °C. A polycarbonate (PC) or PP plate with a thickness of 5 mm was employed as a print bed, with the bed temperature maintained at room temperature (ca. 25 °C). A PP plate as a print bed performed better than PC as higher adhesion is observed for PP.

### Shape Memory

**Figure 6:**
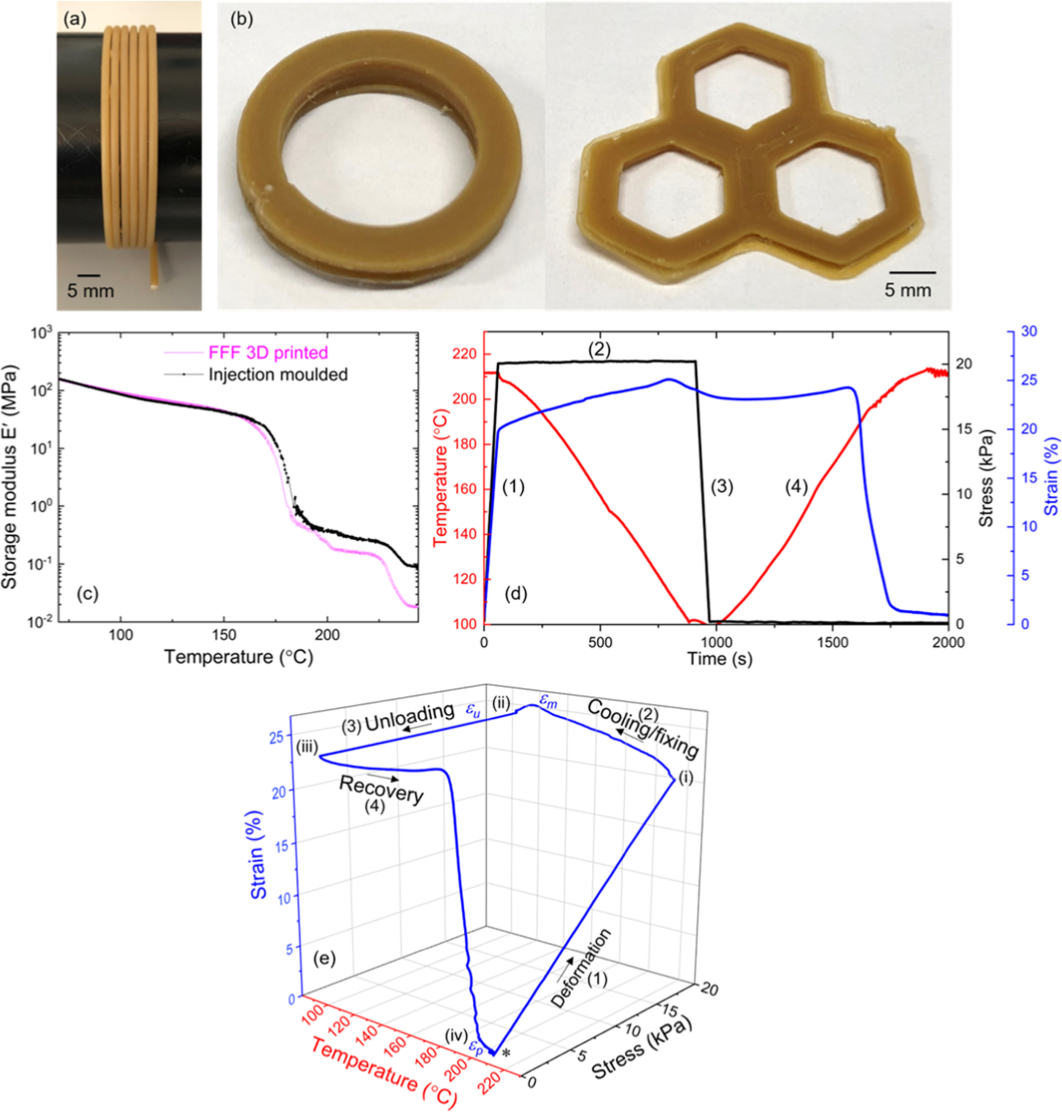
Fused filament fabrication of a dynamically crosslinked network and shape memory. (a) Filament of PP-vit with diameter *D* ≈ 1.75 mm derived from reactive extrusion; (b) various 3D-printed objects; and (c) comparison of the storage modulus of injection-molded and 3D-printed objects, the slightly lower ‘rubber plateau’, and the deeper drop of the elastic–plastic transition are explained by the heterogeneous nature of the printed object made of the thermally extruded filaments. (d) Shape memory in a 3D-printed dynamically crosslinked network: Thermomechanical cycles conducted in DMA for the estimation of fixity and recovery. (1) Deformation: the sample was deformed with a tensile stress of 20 kPa at 210 °C. (2) Cooling/fixing: With the stress maintained at 20 kPa, the sample was then cooled below *T*_c_ (≈70 °C). (3) Unloading: the stress was reduced to 0 kPa. This step fixes the specimen to a temporal shape. Shape fixity (*R*_f_) is calculated at this step. (4) Recovery: the sample was reheated to 210 °C. (e) Evolution of strain in the PP vitrimer with the application of thermomechanical program demonstrating the fixity–recovery cycle.

Quantitative estimation of shape fixing and shape recovery from an intermediate shape to the initial reference shape was conducted with a DMA 850 (TA instrument). Dynamically crosslinked filament extruded from the melt compounder was 3D-printed (FFF) into a rectangular sample and mounted between the clamps of DMA in tensile mode. The instrument was equilibrated at 210 °C for 15 min before starting the experiment (see Figure 6d). A thermomechanical cycle consisting of four steps was applied to the sample. (1) Deformation: The rectangular strip was deformed with the application of stress (20 kPa) at a constant stress ramp of 20 kPa/min at 210 °C. (2) Cooling/fixing: With the maintained stress of 20 kPa, the sample was cooled below *T*_c_ (≈70 °C) at a cooling rate of 10 °C/min to record the stretched shape. (3) Unloading: The stress was reduced to 0 kPa at a rate of 20 kPa/min. This step fixes the specimen to an intermediate shape. Shape fixity (*R*_f_) is calculated at this step. (4) Recovery: The sample was reheated to 210 °C at 10 °C/min and maintained isothermally for 5 min to allow it to recover its natural shape prescribed by crosslinking, before starting the next cycle. Recovery (*R*_r_) of the sample from the intermediate shape to the initial reference shape is calculated after this step. This thermomechanical cycle was repeated several times to evaluate the quality and reproducibility of the shape fixing and recovery. Physical demonstration of the shape-memory effect was performed on a 3D-printed rectangular specimen of dimensions 28 mm × 5 mm × 1 mm. The strip was deformed into a twisted shape at 210 °C and cooled (*T* ≈ 50 °C) under the maintained stress to fix the intermediate shape. The twisted specimen was unloaded. A stress-free reheating of the specimen leads to the recovery of the initial shape (see the videos in the Supporting Information).

## Results and Discussion

### Reactive Extrusion of a Dynamic Network

Polyolefins made of solely carbon–carbon and carbon–hydrogen bonds were chosen as a model system to introduce chemical functionality for the dynamic network. Commercial PP with a moderate melt flow index of 34 g/10 min was our base material. Functionalization through solution chemistry was avoided as PP has very limited solubility in common organic solvents. Moreover, solvent-free chemical functionalization is highly desirable in an industrial setting.^33^ Free radical coupling of maleic anhydride initiated by DCP was adopted to introduce anhydride units in the polymer backbone, producing MA-*g*-PP.^14,34^ A laboratory-scale polymer compounder was employed for the reactive extrusion. Radical coupling was performed under constant N_2_ flow to maintain an inert atmosphere, minimizing radical loss. MA-*g*-PP was subsequently crosslinked with a chemical moiety bearing bifunctional epoxy groups, PBAEG and catalyst Zn(acac)_2_ (cf. Figure 1). The efficiency of MA grafting onto PP was confirmed by FT-IR analysis, as shown in Figure 2. DCP, a free-radical initiator, is needed for MA grafting onto PP. This was proved by a comparison of reactive extrusion of PP with and without the DCP initiator (in the latter case, no MA grafting would occur). After the PP was extruded, it was dissolved in hot xylene and then precipitated in acetone. The FT-IR absorbance band at 1785 cm^–1^ is attributed to the C=O symmetrical stretching. Extrusion of PP and MA without a free radical initiator shows very little absorbance at 1785 cm^–1^ from MA, which disappears altogether after precipitation in acetone, as the free MA readily dissolves in acetone and does not precipitate. When reactive extrusion is done in the presence of DCP, MA is covalently bonded onto PP; hence, even after precipitation in acetone, the absorption band corresponding to the C=O bond is retained. The titration method was adopted to quantitively assess the grafting reaction, estimating the fraction of MA grafting to be 2.2 wt %.

Epoxy–anhydride curing is believed to undergo via anionic ring-opening copolymerization between epoxy and anhydride.^35,36^ The permanent nature of crosslinking in the vitrimer (PP-vit) was proved by the swelling (gel fraction) experiment. After immersing in the “good” solvent xylene at a high temperature (120 °C), an insoluble fraction was recovered. Gel fraction was estimated at ≈ 52%. In stark contrast, the thermoplastic precursor PP was dissolved in hot xylene within 15 min of immersion. The extent of crosslinking primarily depends on the degree of random grafting of MA and the efficiency of anhydride–epoxy curing. In our case, we achieved a gel fraction higher than that recently reported in the literature on polyolefin-based vitrimers with different bond exchange mechanisms.^14^

The melting and crystallization behavior of the crosslinked network was probed through DSC. Crosslinking primarily occurs in the amorphous segment of the semicrystalline polymer, and hence the overall DSC thermogram would not significantly alter.^30^ The heterogeneous microdomain of the crosslinked part serves as a nucleating agent and increases the crystallization temperature (see Figure 3). Crosslinking also serves as a physical barrier to segmental mobility and chain packing, reducing the overall crystallinity. The heat of fusion was calculated from the area under the endothermic curve of the second heating scan in the DSC profile. We found that the crystallinity fraction was reduced by only 3% in the crosslinked network compared to the thermoplastic precursor. Overall, the semicrystalline nature of the precursor thermoplastic was well preserved upon crosslinking. Moreover, a room-temperature stress–strain behavior (see Figure 4) reveals the improvement of tensile strength. The extruded material was ground and injection-molded to prepare samples according to the ASTM D638 standard (see Figure 4a). Compared to the thermoplastic precursor, a slight decrease in the elongation to break is normally observed in vitrimers as the crosslinks act as a barrier in chain extension (Figure 4b). This is an insignificant matter since a ductility of over 100% is still observed. The comparison of the precursor PP and its vitrimer, crosslinked at quite a low density, indicates that they have approximately the same linear (Young) modulus, *E* = 380 MPa, determined by the same polycrystalline microstructure, a slightly higher yield stress (no doubt due to added crosslinking) of 25–26 vs 22–23 MPa in the original PP and at about the same tensile strain of 16–18%. The two tensile curves in Figure 4b represent the just-produced vitrimer and the vitrimer that has been “recycled” five times (chopping into small pieces, recompounding, and injection molding again). We see no degradation of material properties, which is a positive factor promising true multiuse of this plastic.

### Thermomechanical Properties

Thermomechanical properties of the vitrimer and the thermoplastic precursor were investigated through dynamic mechanical analysis (DMA). Temperature-dependent linear storage modulus (*E*′) is shown in Figure 5a. The DMA profile of the samples resembles the typical thermoplastic behavior. At a lower temperature (*T* < *T*_m_), the storage modulus *E*′ is dictated by the semicrystallinity. Since the vitrimer retains the overall semicrystallinity of PP, the modulus is very close to the precursor thermoplastic, until the melting point is reached (which depends on the rate of heating, as indicated in the plot). Beyond the *T*_m_, thermoplastic flows as a viscous liquid. It is also worth noting that the quasi-equilibrium melting temperature obtained at 0.5^o^/min is more accurately captured at a lower heating rate in DMA. In stark contrast, the corresponding dynamically crosslinked network has a nonvanishing *E*′ beyond the *T*_m_, with a signature “rubber plateau”, the hallmark of an elastomeric network. PP-vit showed a rubber modulus of ≈500 kPa, and it fairly remained constant till 220 °C. An important feature of the “rubber plateau” in vitrimers shows as a step drop: at a higher temperature for a higher heating rate. Although one cannot see the true elastic–plastic transition in a dynamic oscillation test (even at a relatively low frequency of 1 Hz), the onset of rapid bond exchange above the “vitrification temperature” is reflected in this step drop in the dynamic modulus in Figure 5a.

Unlike traditional thermosets, the key characteristic of vitrimers is their ability to plastically flow when heated. The rate of bond exchange reaction underpinning the plastic flow increases with temperature. This is demonstrated by the “iso-stress” creep experiment in DMA presented in Figure 5b. The sample was equilibrated at a specific chosen temperature, where the bond exchange was sufficiently activated, and an instantaneous stress was applied. This results in an initial elastic response, determined by the rubber modulus, after which a plastic flow (creep) sets in until the sample finally breaks. At higher temperatures, the measured rate of plastic flow is higher, and the enhancement is rendered by a higher rate of bond reshuffling. Despite being permanently crosslinked, the ability of vitrimers to plastically flow under stress opens the possibility of topological reconfiguration through compression molding, injection molding, and additive manufacturing. The activation energy of transesterification bond exchange^10^ was found to be higher than most other known bond exchanges in vitrimers,^14,37^ which makes our dynamically crosslinked network a good choice for applications where creep at the service temperature and beyond is not desired.^10^ Vitrimers with lower activation energy undergo significant creep in the vicinity and beyond the melting transitions, diminishing their practical relevance.

The melt flow index (MFI) is an essential parameter to determine the processing conditions of plastics. Clearly, our vitrimers are processable, as they are extruded and injection-molded. We measured the MFI of our vitrimers using the ASTM D1238 process and the standard PP parameters. This gave an MFI of 8–10 g/10 min (compared to MFI = 34 g/10 min of the original PP), and recalculating the effective viscosity at 230 °C, we obtain η = 3·10^–9^ Pa·s.

### Fused Filament Fabrication of a Dynamic Network

Engineering applications can be enormously expanded if the fabrication of the smart materials is digitally encoded. With this aim, we wanted to explore the fused filament fabrication (FFF method of 3D printing) with the extruded filament of the dynamically crosslinked polymer (see Figure 6a). The bond exchange reaction above the “vitrification temperature” enables extrusion via the plastic flow and the subsequent 3D printing despite the material being permanently crosslinked. As shown in Figure 6b, objects with various shapes were printed in a commercial 3D printer (see Supporting Information movie S1). The evidence of the network integrity after 3D printing is further demonstrated by comparing with DMA profiles of the as-extruded specimen with the DMA trace of an original injection-molded specimen. Figure 6c shows that the 3D-printed specimen has a slightly lower rubber modulus and a deeper drop at the elastic–plastic transition, but fundamentally the material properties remain the same (especially in the solid plastic state where their main use is expected). In the earlier section, we have shown that a dynamically crosslinked network behaves as a classical SMP, as long as the material is not heated above the “vitrification temperature” under load (in which case the plastic creep would occur).

The enhanced elastic nature of a crosslinked network brings exciting macroscopic properties in response to external stimuli.^38^ We examined the shape-memory behavior of 3D-printed objects. Shape-memory polymers (SMPs) are a class of solid polymers capable of mechanical programming by a thermal stimulus.^39−41^ Broadly, SMPs can “remember” one or more reference shapes, determined by the network formation topology, but can be reconfigured to one or more temporary shapes, with the initial reference shape still recoverable. Depending upon the chemical nature of the polymer network, the stimulus for recovery could be heat and light. In the present case, the bond exchange reaction is thermally triggered. We found that at load-free reheating, the 3D-printed specimen rapidly responds to the thermal stimulus and returns to the reference shape (see Supporting Information movie S2). This further validates the network integrity, and mechanical responsiveness to thermal stimulus is well preserved after fused filament fabrication. The shape-memory response can be quantitatively investigated through a repeated thermomechanical cycle (see Figure 6d). When SMPs are derived from semicrystalline polymers, as is the case with the PP vitrimer, the material is deformed in the rubbery state above the melting temperature. Subsequently, a programmed shape is fixed by cooling below the crystallization temperature, which immobilizes the polymer chains by arresting the segmental motion. Latent strain energy is stored in this deformed state. The recovery is triggered through a “load-free” (with zero applied stress) heating process by bringing the material back into the rubbery state when the stored strain energy is released and the sample returns to the reference shape. The underlying driving force of the recovery is the transition between the network with restricted chain mobility to the state with higher conformational entropy in the rubber elastic regime. The most important aspect of SMPs in a practical scenario and their technological competence is quantified by measuring the quality of shape fixing (*R*_f_, fixity) and the extent of recovery (R_*r*_, recovery), which can be assessed through the DMA thermomechanical cycle, as shown in Figure 6d. The extent of shape fixing is calculated as^41^
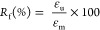
where ε_m_ is the strain achieved after deforming in the second step and ε_u_ is the strain after unloading. The capacity to “remember” the original shape is determined by the extent of recovery. Shape recovery from the programmed shape is obtained through load-free heating. After equilibrating at a high temperature in the rubbery regime, the shape recovery is estimated as^41^
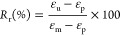
where ε_*p*_ is the strain achieved after the recovery (4th) step and ε_u_ and ε_m_ are the same as earlier. From the data plotted in Figure 6e, these parameters are estimated as *R*_f_ = 92% and *R*_r_ = 90%. This high quality of shape fixing and recovery is making the vitrimer network a good choice for modern technology development with interactive interfaces exploiting the stimuli-responsive nature of SMP.

## Conclusions

Unprecedented usage of nonrenewable petrochemical in making plastics leaves a massive carbon footprint. To curb this rising anthropogenic GHG emission, it is crucial to consolidate an internal mechanism to recover and upcycle high-value materials from commodity plastics after their service life, thereby minimizing the carbon-intensive process of virgin polymer production. We have demonstrated that dynamic covalent chemistry enables thermoplastic materials to turn into high-performance thermosets, retaining mechanical integrity, chemical insolubility, and shape-memory characteristics at the service temperature, yet continually reprocessable using conventional injection molding or 3D printing above the “vitrification temperature.” Although permanently crosslinked, vitrimers undergo creep under stress when the bond exchange is sufficiently activated, giving the underpinning plastic flow. Above the melting transition, a classical rubber plateau is identified, a hallmark of elastomeric materials. Fused filament fabrication of these materials was achieved with excellent dimensional accuracy. The 3D-printed specimen not only shows mechanical integrity but also a high level of responsiveness to a thermal stimulus. The thermomechanical cycle employed in dynamic mechanical analysis reveals that shape fixation and shape recovery from the programmed state is well over 90%. Moreover, orthogonality of the bond exchange reaction with a variety of additives and unidentified contaminants makes the near-pristine inputs unnecessary, allowing as-received recycled plastic to be converted to vitrimers. Thus, our work lays a foundation to reclaim megatons of postconsumer thermoplastics, converting them into technically and economically valuable materials.

## Supporting Information Available

Video 1: Fused filament fabrication (3D printing) of a dynamically crosslinked network (MP4)
Video 2: Thermally triggered recovery of 3D-printed shape-memory polymer (MP4)
